# To examine the associations between medical students’ conceptions of learning, strategies to learning, and learning outcome in a medical humanities course

**DOI:** 10.1186/s12909-019-1856-8

**Published:** 2019-11-08

**Authors:** Yu-Chun Chiu, Jyh-Chong Liang, Hong-Yuan Hsu, Tzong-Shinn Chu, Kuan-Han Lin, Yen-Yuan Chen, Chin-Chung Tsai

**Affiliations:** 1 Department of Pediatrics, National Taiwan University Hospital, #7, Rd. Chong-Shan S, Taipei, 10002 Taiwan; 2 Department of Medical Education, National Taiwan University Hospital, #7, Rd. Chong-Shan S, Taipei, 10002 Taiwan; 3 Program of Learning Sciences, National Taiwan Normal University, #162, Rd. Heping E. Sec. 1, Taipei, 10610 Taiwan; 4 Graduate Institute of Medical Education & Bioethics, National Taiwan University College of Medicine, #1, Rd. Ren-Ai sec. 1, Taipei, 10051 Taiwan; 5 Department of Internal Medicine, National Taiwan University Hospital, #7, Rd. Chong-Shan S, Taipei, 10002 Taiwan; 6 Department of Healthcare Administration, Asia University, #500, Rd. Liou-Feng, Wufeng, Taichung, 41354 Taiwan

**Keywords:** Conceptions of learning, Approaches to learning, Strategies to learning, Medical humanities

## Abstract

**Background:**

By learning medical humanities, medical students are expected to shift from handling the diseases only to seeing a whole sick person. Therefore, understanding medical students’ learning process and outcomes of medical humanities becomes an essential issue of medical education. Few studies have been conducted to explore factors surrounding medical students’ learning process and outcomes of medical humanities. The objectives were: (1) to investigate the relationships between medical students’ conceptions of learning and strategies to learning; and (2) to examine the relationships between students’ strategies to learning and learning outcomes for medical humanities.

**Methods:**

We used the modified Approaches to Learning Medicine (mALM) questionnaire and Conceptions of Learning Medicine (COLM) questionnaire to measure the medical students’ strategies to learning and conceptions of learning respectively. The learning outcome of medical humanities was measured using students’ weighted grade in a medical humanities course. The confirmatory factor analysis (CFA) was used to validate the COLM and mALM questionnaires, in which construct validity and reliability were assessed. Pearson’s correlation was used to examine the relationships among the factors of COLM, mALM, and the weighted grade. Path analysis using structural equation modeling technique (SEM) was employed to estimate the structural relationships among the COLM, mALM, and the weighted grade.

**Results:**

Two hundred and seventy-five first-year medical students consented to participate in this study. The participants adopting surface strategies to learning were more likely to have unsatisfactory learning outcome (β = − 0.14, *p* = .04). The basic-level conception of “Preparing for Testing” was negatively (β = − 0.19, *p* < .01) associated with deep strategies of learning, and positively (β = 0.48, *p* < .01) associated with surface strategies of learning (β = 0.50, *p* < .01). The basic-level conception of “Skills Acquisition” was positively associated with deep strategies of learning (β = 0.23, *p* < .01).

**Conclusion:**

Medical educators should wisely employ teaching strategies to increase students’ engagement with deep and self-directed learning strategies, and to avoid using surface learning strategies in the medical humanities course in order to achieve better learning outcomes.

## Background

Medical humanities is a multidisciplinary field including humanities, social sciences, the arts, and their applications to clinical practice [1, 2]. By learning medical humanities, medical students are expected to think critically, to understand personal values, and to be equipped with cultural competence, leadership and teamwork, and empathy. Given that medical humanities is considered as shifting medicine from handling the diseases only to seeing a whole sick person [3], and that learning medical humanities is expected to prepare medical students for responding appropriately to complex clinical contexts [4], there has been a consensus that medical humanities should be integrated into the medical curriculum [5]. Although the importance of medical humanities is usually highlighted in medical education, medical humanities remained as the undesirable part of medical education, and medical humanities curriculum in medical schools receives more critiques than praises [2, 6]. Therefore, understanding medical students’ learning process and learning outcomes of medical humanities courses becomes an essential issue of medical education.

The learning process and outcomes of medical students have always been greatly concerned about by their teachers and medical educators, particularly the learning outcomes of medical humanities. Better learning outcomes for medical humanities imply that medical students may be capable of responding to complex clinical context. Among the factors which influence learning outcomes, conceptions of learning and approaches to learning have been reported as two of the most influential factors on students’ learning [7–11].

“Conceptions of learning” is defined as learners’ coherent knowledge and beliefs about the whole picture of learning [7, 12]. Tsai reported two levels of conceptions of learning, and each level includes a total of three to four factors, i.e. higher-level conceptions of learning (“Increasing One’s Knowledge”, “Applying”, “Understanding”, and “Seeing in a New Way”) and lower-level conceptions of learning (“Memorizing”, “Testing”, “Calculating”, and “Practicing Tutorial Problems”) [7]. In comparison, “approaches to learning” implies students’ motivations and strategies to learn or process the academic work [13]. Prior studies have reported surface approaches and deep approaches to learning. Surface approaches to learning include surface motivations (“Fear of Failure” and “Aim for Qualification”) and surface strategies (“Minimizing Scope of Study” and “Memorization”), and deep approaches to learning include deep motivations (“Intrinsic Interest” and “Commitment to Work”) and deep strategies (“Relating Ideas” and “Understanding”) [11, 14, 15].

Several studies have examined the relationships between conceptions of learning and approaches to learning in the disciplines of science [16], biology [11], and computer science [17]. Students with higher-level conceptions of learning utilized deep strategies to learning, and those with lower-level conceptions utilized surface strategies [11, 16]. Both lower-level and higher-level conceptions of learning have been found to be positively associated with surface motivation in computer science [17]. Prior studies in medical education have been mostly focused on the approaches to learning [18–22], but few of them were focused on the conceptions of learning and the relationship between the conceptions of learning and the approaches to learning in medical students.

Previous studies have reported the relationships between the approaches to learning and learning outcomes for non-medical learners [23, 24]. For example, Snelgrove et al. reported that student nurses’ deep approaches to learning sociology were positively and significantly related to their exam results and grade point average, and surface strategies negatively related to the learning outcomes without statistical significance [23]; and Chamorro-Premuzic et al. also found that the use of the deep approaches to learning was the most influential than personality and intelligence to account for the variances of students’ academic performance [24]. In addition, the relationships between the approaches to learning and the learning outcomes for medical leaners were also examined [18, 22, 25]. For example, Reid et al. showed that medical students scored positively with deep approaches to learning and negatively with surface approaches [25]. Liang et al. also examined the relationships between the approaches to learning and learning outcomes. They reported that the deep strategy, i.e. relating ideas and understanding, significantly predicted better learning outcomes, and the surface strategy, i.e. minimizing scope of study, significantly predicted unsatisfactory learning outcomes [22].

Although higher-level and lower-level conceptions of learning, and approaches to learning (deep motives, surface motives, deep strategies, and surface strategies) were studied in medical education, none of the prior studies have been conducted to examine those surrounding the learning outcomes of medical humanities. Accordingly, this study aimed to investigate the relationships between medical students’ conceptions of learning and strategies to learning, and to examine the relationships between students’ strategies to learning and learning outcomes for medical humanities.

## Methods

### Setting

We conducted this study in the most academically prestigious medical school in a university located in Northern Taiwan. While this study was conducted, the medical school enrolled approximately 155 students each year.

### Data collection

We recruited the first-year medical students of the 2015—2016 cohort and the 2016—2017 cohort to participate in this study, all of whom had taken the required course of “Medicine and Humanities.”

This course was mainly composed of four segments general introduction: medical arts, medical history, medical philosophy, and Taiwan literature. A group of lecturers participated in teaching different topics in this lecture-based medical humanities course. Each topic, belonging to one of the four segments, was a two-hour lecture. The two-hour lecture was conducted once every week, 15–16 lectures a semester. In addition, students were required to participate in an experiential learning activity, and to give feedback and reflections on their learning.

The students were asked whether they would like to participate in this study or not. Two questionnaires with a cover letter explaining the purpose of this study were distributed to those who consented to participate in this study. In addition, the weighted grade of each participating medical student of the “Medicine and Humanities” course was considered the learning outcome of medical humanities.

### Instruments

We measured the first-year medical students’ conceptions of learning medicine using the Conceptions of Learning Medicine (COLM) questionnaire, which originally had seven developed [7] factors. Two factors, “Skills Acquisition” and “Communication”, were added thereafter, using the contributions from three medical professionals, with the aim to demonstrate the unique characteristics of the medical discipline.

The COLM questionnaire used in this study consists of nine factors: advanced-level conceptions of learning medicine, highlighting relating learning medicine to application to medical practice, including “Increasing One’s Knowledge”, “Applying”, “Understanding”, “Seeing in a New Way”, and “Communication”; and basic-level conceptions of learning medicine, focusing on learning medicine itself without paying attention to application to medical practice, including “Memorizing”, “Preparing for Testing”, “Practicing Tutorial Problems”, and “Skills Acquisition.” Each factor contains a total of five to seven items, and a total of 56 items are included in the COLM questionnaire. A participant’s score of each factor was calculated by taking the average of all items in the factor. The sample items for each factor included in advanced-level and basic-level conceptions of learning medicine are shown below (Appendix 1):

Basic-level COLM: Memorizing

“Learning medicine means memorizing the physiological mechanisms of humans in medical textbooks”

Basic-level COLM: Preparing for Testing

“Learning medicine means passing all the examinations to obtain professional certification”

Basic-level COLM: Practicing Tutorial Problems.

“Learning medicine means practicing with a SimMan simulator.”

Basic-level COLM: Skills Acquisition.

“Learning medicine means learning how to study systematically, such as using a concept map.”

Advanced-level COLM: Increasing One’s Knowledge.

“Learning medicine means acquiring more medical knowledge.”

Advanced-level COLM: Applying

“Learning medicine means solving human medical problems.”

Advanced-level COLM: Understanding

“The purpose of learning medicine is to understand medical knowledge.”

Advanced-level COLM: Seeing in a New Way

“Learning medicine means expanding my medical knowledge and visions.”

Advanced-level COLM: Communication

“Learning medicine means learning how to cooperate with others as a team to complete the task.”

We used the modified Approaches to Learning Medicine (mALM) questionnaire, borrowing from the Approaches to Learning Medicine (ALM) questionnaire, for measuring the first-year medical students’ strategies to learning medicine [22]. This mALM questionnaire is composed of deep strategies (four items for “Relating Ideas” and four items for “Understanding”) and surface strategies (seven items for “Minimizing Scope of Study” and four items for “Memorization”). All the items were coded using a Likert scale ranging from one to five, representing “strongly disagree” to “strongly agree,” respectively. A participant’s score of each strategy was calculated by taking the average of a factor’s all included items in the strategy. The sample items for each strategy included in deep strategies and surface strategies are shown below (Appendix 2):

Deep Strategies: Relating Ideas

“When learning medicine, I like to create a new plausible theory for helping me to summarize a lot of disorganized content.”

Deep Strategies: Understanding

“When learning medicine, I try to understand the content of the medical courses.”

Surface Strategies: Minimizing Scope of Study

“When learning medicine, I spend as little time on studying medicine as I can, as long as I feel that I can pass the exams. There are many other interesting things to do.”

Surface Strategies: Memorization

“When learning medicine, I am very focused on those which will be tested in exams, and I memorize them by rote.”

The learning outcome of medical humanities was measured using students’ weighted grade in the medical humanities course. The weighted grade was based on a global rating consisting of 40% class participation, 25% short paper writing surrounding medical humanities issues, 25% term examination using multiple choice questions focused on the lecture and assigned readings of the course, and 10% motivation and performance in experiential learning activities. Students’ grades were collected at the end of the course, and the score was treated as a continuous variable ranging from 0 to 100.

### Statistical analysis

The confirmatory factor analysis (CFA) was used to validate the COLM and mALM questionnaires, in which construct validity and reliability were assessed. The factor loading of each item, average variance explained (AVE), and composite reliability (CR) were estimated.

Pearson’s correlation was used to examine the relationships among the factors of COLM, mALM, and weighted grade. The relationships between two variables/factors with a *p* value of less than .20 were retained for further path analysis.

Path analysis using structural equation modeling technique (SEM) was employed to estimate the structural relationships among the COLM, mALM, and students’ learning outcomes. The goodness of model fit was assessed, using the goodness of fit index (GFI), comparative fit index (CFI), and root-mean-square error of approximation (RMSEA), and normed fit index (NFI), to ensure that the structural model reasonably explained the structural relationships among COLM, mALM, and learning outcomes.

A *p* value of less than .05 were considered statistically significant. All statistical analysis was carried out using SPSS AMOS 24 software (IBM Corp., Armonk, NY, USA). This study was approved by the Social and Behavioral Research Ethics Committee in National Taiwan University (201505HS002). The first-year medical students were asked verbally of their preference to participate in this study or not. Written consent was then obtained from those who preferred to participate in this study by signing the informed consent form, which was approved by the Social and Behavioral Research Ethics Committee in National Taiwan University.

## Results

Two hundred and seventy-five (97.52%) of the 282 first-year medical students, 130 from the 2015—2016 cohort and 145 from the 2016—2017 cohort, consented to participate in this study. Among those 275 participating first-year medical students, 272 (98.91%), 67 females (24.63%) and 205 males (75.37%), completely answered the two questionnaires and were eligible for data analysis. The participants’ ages ranged from 17.08 to 30.22 years old, with an average of 19.30 (standard deviation = 1.64).

By CFA, 38 items, belonging to the four factors of advanced-level conceptions and five factors of basic-level of conceptions, were retained in the final version of the COLM questionnaire. The results of CFA revealed significant factor loadings for all items (values larger than 0.5) [26]. Furthermore, the scores of AVE and CR for the nine factors were higher than the threshold values of 0.5 and 0.7 [27, 28], with the scores ranging from 0.61 to 0.82 and 0.86 to 0.95, respectively, indicating acceptable construct validity and reliability (Table 1).

**Table 1:** The CFA analysis for the Conception of Learning Medicine (COLM) questionnaire. (*N* = 272)

Construct and Measurement Items	Factor loading	t Statistics (*p* Value)	CR	AVE	Alpha Value
Memorizing (M) Mean = 2.17, SD = 0.84	–	–	0.86	0.61	0.86
M 3 (3)	0.79	–	–	–	–
M 4 (4)	0.80	13.44 *p* < .01	–	–	–
M 5 (5)	0.76	12.72 *p* < .01	–	–	–
M 6 (6)	0.78	13.10 *p* < .01	–	–	–
Preparing for Testing (PT) Mean = 0.90, SD = 0.75	–	–	0.87	0.70	0.84
PT 3 (9)	0.80	–	–	–	–
PT 4 (10)	0.78	12.65 *p* < .01	–	–	–
PT 6 (12)	0.81	12.96 *p* < .01	–	–	–
Practicing Tutorial Problems (PTP) Mean = 3.01, SD = 0.58	–	–	0.90	0.70	0.81
PTP 2 (15)	0.54	–	–	–	–
PTP 4 (17)	0.75	8.49 *p* < .01	–	–	–
PTP 5 (18)	0.84	8.95 *p* < .01	–	–	–
PTP 6 (19)	0.81	8.83 *p* < .01	–	–	–
Increasing One’s Knowledge (IOK) Mean = 3.02, SD = 0.54	–	–	0.92	0.71	0.85
IOK 2 (21)	0.63	–	–	–	–
IOK 4 (23)	0.84	11.02 *p* < .01	–	–	–
IOK 5 (24)	0.84	11.03 *p* < .01	–	–	–
IOK 6 (25)	0.72	9.90 *p* < .01	–	–	–
IOK 7 (26)	0.64	9.01 *p* < .01	–	–	–
Applying (A) Mean = 3.31, SD = 0.50	–	–	0.92	0.69	0.82
A 1 (27)	0.74	–	–	–	–
A 2 (28)	0.79	12.37 *p* < .01	–	–	–
A 3 (29)	0.75	11.76 *p* < .01	–	–	–
A 5 (31)	0.63	9.92 *p* < .01	–	–	–
A 7 (33)	0.57	8.90 *p* < .01	–	–	–
Understanding (U) Mean = 3.14, SD = 0.50	–	–	0.93	0.78	0.84
U 1 (34)	0.75	–	–	–	–
U 2 (35)	0.85	13.51 *p* < .01	–	–	–
U 3 (36)	0.78	12.54 *p* < .01	–	–	–
U 5 (38)	0.63	10.08 *p* < .01	–	–	–
Seeing in a New Way (SNW) Mean = 3.06, SD = 0.64	–	–	0.93	0.73	0.89
SNW 2 (41)	0.78	–	–	–	–
SNW 3 (42)	0.79	13.44 *p* < .01	–	–	–
SNW 4 (43)	0.83	14.23 *p* < .01	–	–	–
SNW 5 (44)	0.81	13.83 *p* < .01	–	–	–
SNW 6 (45)	0.72	12.21 *p* < .01	–	–	–
Skills Acquisition (SA) Mean = 3.25, SD = 0.63	–	–	0.93	0.82	0.87
SA 3 (48)	0.86	–	–	–	–
SA 4 (49)	0.92	17.58 *p* < .01	–	–	–
SA 6 (51)	0.73	13.69 *p* < .01	–	–	–
Communication (C) Mean = 3.39, SD = 0.60	–	–	0.95	0.80	0.91
C 1 (52)	0.70	–	–	–	–
C 2 (53)	0.90	13.97 *p* < .01	–	–	–
C 3 (54)	0.90	14.02 *p* < .01	–	–	–
C 4 (55)	0.87	13.57 *p* < .01	–	–	–
C 5 (56)	0.73	11.56 *p* < .01	–	–	–

Abbreviation List: *CFA* Confirmatory Factor Analysis, *CR* Composite Reliability, *AVE* Average Variance Extracted, *M* Memorizing, *PT* Preparing for Testing, *PTP* Practicing Tutorial Problems, *IOK* Increasing One’s Knowledge, *A* Applying, *U* Understanding, *SNW* Seeing in a New Way, *SA* Skills Acquisition, *C* Communication

**Table 2:** The CFA analysis for the modified Approaches to Learning Medicine (mALM) questionnaire. (*N* = 272)

Construct and Measurement Items	Factor loading	t Statistics (*p* Value)	CR	AVE	Alpha Value
Deep Strategy (DS) Mean = 3.07, SD = 0.52	–	–	0.95	0.79	0.88
DS 1 (1)	0.68	–	–	–	–
DS 3 (3)	0.91	13.34 *p* < .01	–	–	–
DS 4 (4)	0.92	13.48 *p* < .01	–	–	–
DS 5 (5)	0.78	11.71 *p* < .01	–	–	–
DS 7 (7)	0.62	9.54 *p* < .01	–	–	–
Surface Strategy (SS) Mean = 1.88, SD = 0.75	–	–	0.77	0.52	0.73
SS 2 (10)	0.75	–	–	–	–
SS 3 (11)	0.75	8.88 *p* < .01	–	–	–
SS 4 (12)	0.59	7.88 *p* < .01	–	–	–

Abbreviation List: *CFA* Confirmatory Factor Analysis, *CR* Composite Reliability, *AVE* Average Variance Extracted, *DS* Deep Strategy, *SS* Surface Strategy

A total of eight items, five for deep strategies and three for surface strategies, were retained in the final version of mALM questionnaire after CFA. The factor loading values ranged from 0.59 to 0.92 and were greater than 0.5, suggesting suitable factor loadings [26]. The values of AVE and CR for two factors of mALM ranged from 0.52 to 0.79 and 0.77 to 0.95, respectively, demonstrating acceptable construct validity and reliability (Table 2) [27, 28].

Table 3 shows the Pearson’s correlation coefficients among the factors of COLM, mALM, and the weighted grade. All factors of the advanced-level conceptions of learning medicine (“Increasing One’s Knowledge”, “Applying”, “Understanding”, “Seeing in a New Way”, and “Communication”) were positively related to the deep strategies to learning medicine (r = 0.13~0.28, *p* values = <.01~.03). In comparison, most of the factors of the basic-level conceptions of learning medicine (“Memorizing”, “Preparing for Testing”, and “Skills Acquisition”) were significantly related to the surface strategies to learning medicine (r = − 0.13~0.43, *p* values = <.01~.03). Interestingly, the medical students with the conception of learning medicine—“Practicing Tutorial Problems” were more likely to learning medical humanities using deep strategies (*r* = 0.16, *p* value = .01).

**Table 3:** Pearson’s correlation coefficient between the Conceptions of Learning and Strategies to Learning, and between the weighted grade and Strategies to Learning Medicine

	Weighted Grade	Advanced-level COLM					Basic-level COLM			
		IOK	A	U	SNW	C	M	PT	PTP	SA
Deep Strategy	0.11 (*p* = .08)	0.13 (*p* = .03)	0.14 (*p* = .02)	0.18 (*p* < .01)	0.16 (*p* = .01)	0.28 (*p* < .01)	−0.06 (*p* = .33)	− 0.23 (*p* < .01)	0.16 (*p* = .01)	0.30 (*p* < .01)
Surface Strategy	−0.13 (*p* = .03)	0.02 (*p* = .73)	−0.06 (*p* = .30)	−0.03 (*p* = .62)	−0.07 (*p* = .28)	−0.13 (*p* = .03)	0.24 (*p* < .01)	0.43 (*p* < .01)	0.09 (*p* = .14)	−0.13 (*p* = .03)

Abbreviation: *COLM* Conceptions of Learning Medicine, *IOK* Increase One’s Knowledge, *A* Applying, *U* Understanding, *SNW* Seeing in a New Way, *C* Communication, *M* Memorizing, *PT* Preparing for Testing, *PTP* Practicing Tutorial Problems, *SA* Skills Acquisition

**Fig. 1:**
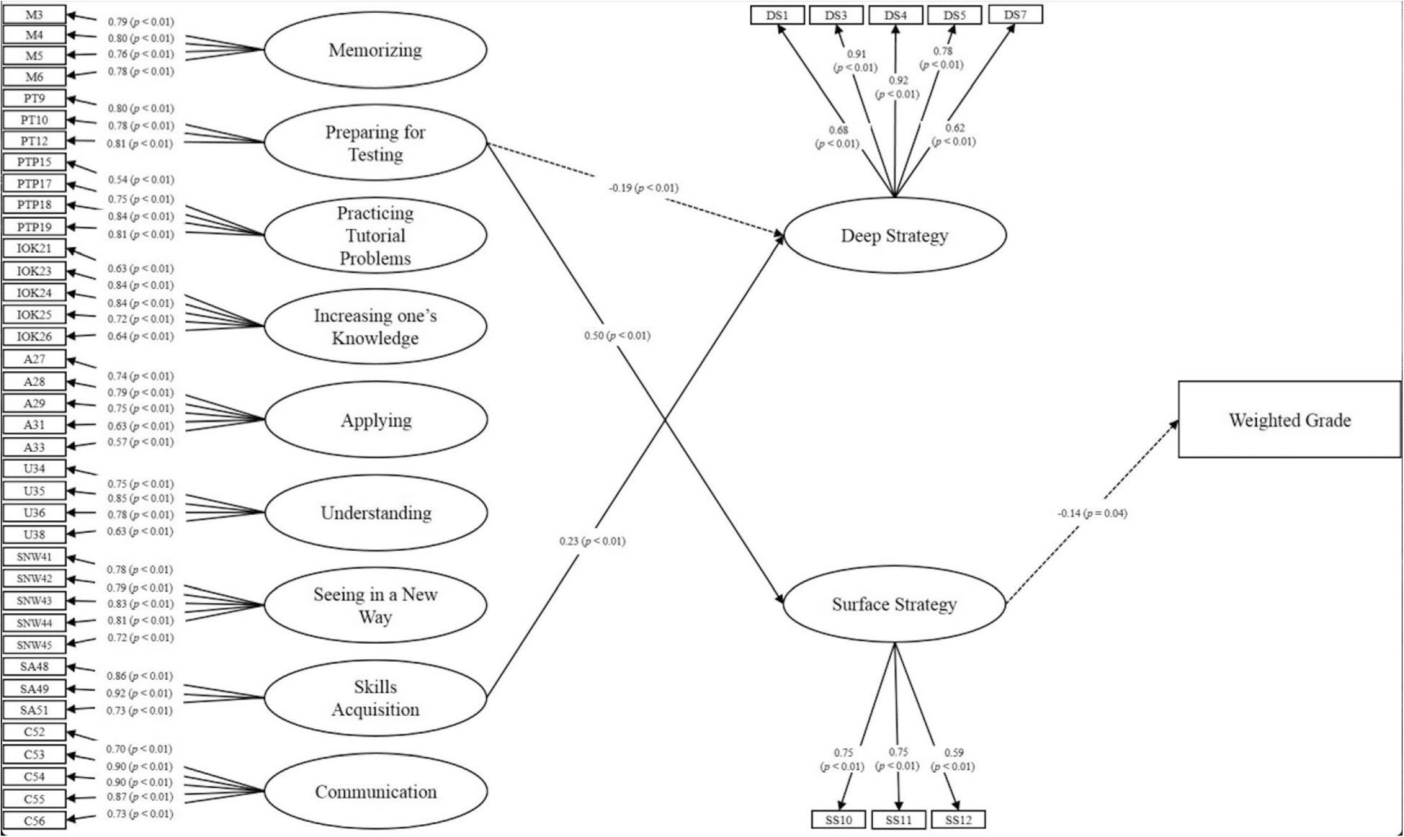
Diagram of structural equation model of the relationships between conceptions of learning, strategies to learning, and learning outcomes

Figure 1 shows the SEM structural model, and only the significant standardized path coefficients are displayed. According to the results of path analysis, the participants with surface strategies to learning medicine were more likely to have worse learning outcome as indicated by the weighted grade of the medical humanities course (β = − 0.14, *p* value = .04). In addition, the basic-level conception of “Preparing for Testing” was negatively (β = − 0.19, *p* value < .01) associated with deep strategies of learning medicine, and positively (β = 0.48, *p* value < .01) associated with surface strategies (β = 0.50, *p* value < .01). The basic-level conception of “Skills Acquisition” was positively associated with deep strategies of learning medicine (β = 0.23, *p* value < .01). The indices suggested an acceptable model fit of the structural model (GFI = 0.80, CFI = 0.72, RMSEA = 0.05, NFI = 0.90) [28, 29].

## Discussion

### The conceptions of and strategies to learning medicine

One of the findings in our study was that medical students holding “Preparing for Testing” COLM, was positively associated with adopting surface strategies to learning, and negatively associated with adopting deep strategies to learning. Similar to our study results, previous studies found that undergraduate students holding lower-level conception of learning tended to employ surface strategies to learning [11, 16]. Liang et al. also reported that computer science-major undergraduate students with lower-level conceptions of learning tended to employ surface strategies to learning computer science [17]. These results imply that students with lower-level conceptions of learning (“Memorizing”, “Preparing for testing”, “Practicing Tutorial Problems”, and “Skills Acquisition”) aimed at adopting surface approaches (the motives of “Fear of Failure” and “Aim for Qualification”, and the strategies of “Minimizing Scope of Study” and “Memorization”) to learning knowledge.

The other interesting finding was that medical students with “Skills Acquisition”, as one of the basic-level conceptions of learning, was positively associated with using deep strategies to learning. The finding contradicts several previous studies pointing out that basic-level conceptions of learning were associated with surface strategies to learning [11, 16, 17].

An explanation may account for this finding. Starting from the first day of receiving medical education, medical students are expected to learn medical knowledge, to acquire clinical skills and to cultivate professional attitudes [30]. Assessments of clinical competencies, such as observing senior physicians’ professional behaviors in clinical encounters, discussion of clinical cases, and feedback from multiple sources, are necessary and important [31]. Successful physicians combine learning medical knowledge, acquiring clinical skills and cultivating professional attitudes with the flexibility required to implement competencies in the clinical encounter in which clinical contexts may change. Therefore, deep approaches to learning, including deep motivation and deep strategies, may maximize learning outcomes for learning medical knowledge, acquiring clinical skills and cultivating professional attitudes. As a result, medical students holding the conception of learning medicine as “Skills Acquisition” may encourage their deep strategies to learning medicine for a better learning outcome.

The other explanation for accounting for this result should refer to the items of “Skills Acquisition” in the COLM questionnaire (Appendix 1). After factor analysis, only three (SA 3, SA 4 and SA 6) of the five items representing “Skills Acquisition” were included in the following SEM model. Obviously, two of the three items (SA 3 and SA 4) clearly relate learning medicine to applying the skills they learned to medical practice, implying that “Skills Acquisition” may be a factor of advanced-level COLM. Therefore, the participants who got a higher score in “Skills Acquisition”, potentially relating learning medicine to application to medical practice as indicated by SA 3 and SA 4, may use deep strategies to learning medical humanities. In addition, this finding also highlights that relocation of “Skills Acquisition” to be a factor of advanced-level COLM, and the items of “Skills Acquisition” used in the COLM questionnaire should be further deliberated. If “Skills Acquisition” is to mean the skills of learning medicine, it should remain in basic-level COLM. In comparison, if “Skills Acquisition” is to mean that learning medicine is to learn the skills applied to medical practice, it should belong to advanced-level COLM. The items of “Skills Acquisition” should be modified accordingly.

Medicine, different from other disciplines such as biology, chemistry, or physics, particularly emphasizes the application of knowledge. However, unlike previous studies [11, 16], our results did not show the significant association between the advanced-level conception of “Applying” and strategies to learning. The insignificant association in this study may be attributed to the dual perspective of the “Applying” concept [7, 16]. On one hand, “Applying” implied that applying knowledge is based on knowing how to use the knowledge [32], and hence is considered as basic-level conception of learning. On the other hand, “Applying” can be interpreted as applying knowledge to practical situation, and thus categorized into advanced-level conception of learning [7]. Potentially due to the first-year medical students’ dual perspective of “Applying”, the association between “Applying” and the strategies to learning may not be significant. In addition, the first-year medical students in Taiwan at this point have not yet immersed themselves to basic or clinical medicine, thus have not yet developed their conception of learning medicine about “Applying”. As a result, the association between “Applying” and the strategies to learning medicine cannot be determined.

### The strategies to learning medicine and learning outcomes in medical humanities

Several previous studies have reported that surface approaches to learning were negatively associated with learning outcomes [18, 25, 33]. Newble et al. also showed that medical students adopting surface approaches to learning, including surface motives and surface strategies mainly learned by rote memorizing and reproducing the material [34]. Liang et al. found that the house officers with surface strategies to learning medicine were more likely to get unsatisfactory learning outcomes [22]. There are two reasons to account for this phenomenon:

Firstly, medical humanities is considered far more multidisciplinary than medicine. Medical students’ approaches to learning did not significantly change in different clinical rotations [35] partly because medical students may see medicine as a single field. In comparison, medical humanities is composed of a variety of disciplines. Rote-like learning for memorizing the knowledge of some of the disciplines may be necessary for integrating it into medical training. Secondly, because medical humanities remained an unappealing part of medical education [2, 6], it is common that medical students, usually with adaptive learning strategies and motivations [22], employ surface strategies for learning medical humanities which is considered unappealing in medical curriculum.

Previous studies pointed out that deep strategies and motivations to learning were a predominant factor affecting medical students’ academic performance [18, 20, 21]. Interestingly, our results did not echo previous studies on the significant association between deep strategies to learning medicine and learning outcomes. One possible reason is that using medical students’ weighted grades in the medical humanities course as a learning outcome may not entirely reflect its association with deep strategies to learning. The other possible reason is that the medical humanities course in this study actually did not induce deep strategies to learning. Many students criticized that medical humanities courses cannot directly provide them with tangible skills that are useful in clinical practice, and hence see them as unappealing [2]. Accordingly, medical students tend to avoid using deep strategies for learning medical humanities which is considered unappealing and useless in clinical practice.

### Strengths and limitations

Previous research has reported close relationships between students’ conceptions of learning and approaches to learning. Nevertheless, few of them have been focused on examining medical students’ learning process and learning outcomes in medical humanities. Our study firstly examined the conceptions of learning, the strategies of learning, and the learning outcomes in medical humanities. Furthermore, we used SEM which is with great strength to provide a summary evaluation of mathematical models involving a lot of linear equations. Nevertheless, several limitations might confine the academic merits of the study results.

First, this was a single-center study, and only first-year medical students were included. In addition, the medical students enrolled in this medical school have done the best in the Advanced Subjects Exam (also known as the University Entrance Exam) or the General Scholastic Ability Test as compared to those enrolled in other medical schools in Taiwan. Accordingly, the generalizability of the study results to other medical students may not be convincing.

Second, several potential factors, such as personal characteristics factors, social and cultural factors, were not included in the structural model. Consequently, the structural model could only explain the differences in medical students’ conceptions of learning medicine and strategies to learning medicine.

Third, the medical students’ learning outcome for medical humanities was measured using weighted grades including a variety of assessments on class participation, written examination, feedback on experiential learning, and a term paper. However, weighted grades in the medical humanities course only represented the medical students’ overall learning performance of medical humanities, which may not represent students’ actual self-directed learning progress (e.g. engagement in extra-curricular activities). Future research should examine the structural model in different indicators for learning outcomes of medical humanities.

## Conclusions

Although the importance of incorporating medical humanities to both undergraduate and post-graduate medical education is continuously emphasized, medical humanities remained an unappealing part in medical curriculum. This study showed that a medical student’s conceptions of learning, such as preparing for testing and skills acquisition, were significantly associated with the strategies to learning medicine, and medical students’ learning outcomes in the medical humanities course were inversely associated with the surface strategies to learning. Therefore, medical educators should wisely employ teaching strategies to increase students’ engagement with deep and self-directed learning strategies, and to avoid using surface learning strategies in the medical humanities course in order to achieve better learning outcomes. By achieving better learning outcomes in medical humanities, medical students are expected to shift from only handling the diseases to seeing a whole sick person, and responding appropriately to complex clinical contexts. Future research is suggested to investigate medical students’ learning process and learning outcome surrounding medical humanities using a larger sample of medical students.

## Data Availability

The dataset used and/or analyzed for this study is available from the co-corresponding authors, Dr. Kuan-Han Lin and Dr. Yen-Yuan Chen, by qualified researchers on a reasonable request.

## Appendix 1

**Table 4:** The Conceptions of Learning Medicine (COLM) questionnaire

	No.	Items
M 1	1	Learning medicine means memorizing the physiological mechanisms of humans in medical textbooks.
M 2	2	Learning medicine means memorizing the proper nouns or important concepts in medical textbooks.
M 3	3	Learning medicine means memorizing what the teacher lectures about in the class.
M 4	4	Learning medicine means memorizing medical symbols, medical concepts or facts.
M 5	5	The most important aspect of learning medicine is to memorize what the teacher taught by reading the collaborative class notes.
M 6	6	The most important aspect of learning medicine is to memorize by repetitively rote and/or mnemonic techniques.
PT 1	7	Learning medicine means passing all the examinations to obtain professional certification.
PT 2	8	Learning medicine means correctly answering the questions in the examinations.
PT 3	9	If there is no examination, I will not prepare for the medical courses.
PT 4	10	There are no benefits to learning medicine except getting high scores on the examinations. In fact, I can get along well without knowing many medical facts.
PT 5	11	The major purpose of learning medicine is to get more familiar with problems and questions in the test.
PT 6	12	I learn medicine for passing the medical examinations.
PT 7	13	The major purpose of learning medicine is to prepare for the future National Licensing Examination.
PTP 1	14	Learning medicine means practicing with a SimMan simulator.
PTP 2	15	Learning medicine means continuously learning in clerkship and internship.
PTP 3	16	After I am done with practice and training in clerkship and/or internship, I expect to have good clinical performance.
PTP 4	17	Learning medicine means knowing how to practice and conduct the correct approach to solving medical problems.
PTP 5	18	Learning medicine, becoming acquainted with the operation, and repetitive practice are closely related.
PTP 6	19	Practicing or training is important because it helps me to integrate medical knowledge.
IOK 1	20	Learning medicine means acquiring more medical knowledge.
IOK 2	21	Learning medicine means acquiring the medical knowledge that I did not know before.
IOK 3	22	I am learning medicine when the teacher teaches me the medical knowledge that I did not know before.
IOK 4	23	Learning medicine means acquiring medical knowledge about how a human body works.
IOK 5	24	Learning medicine helps me acquire medical knowledge about diseases.
IOK 6	25	Learning medicine means increasing my medical knowledge of a human body.
IOK 7	26	Learning medicine means acquiring new medical knowledge.
A 1	27	Learning medicine means solving human medical problems.
A 2	28	Learning medicine means acquiring medical knowledge and skills to solve the medical problems which happen in real life.
A 3	29	Learning medicine means acquiring medical knowledge and skills to enhance the quality of our lives.
A 4	30	The purpose of learning medicine is learning how to apply medical methods I already know to medical problems which I haven’t encountered.
A 5	31	Learning medicine means learning how to apply medical knowledge and skills I already know to solve patients’ medical problems.
A 6	32	We learn medicine to make human lives healthier and more convenient.
A 7	33	Learning medicine means solving or treating diseases.
U 1	34	The purpose of learning medicine is to understand medical knowledge.
U 2	35	Learning medicine means understanding the relationship between different medical concepts.
U 3	36	Learning medicine means understanding a variety of treatments.
U 4	37	Learning medicine means an understanding of medical problems and phenomena that I did not know before.
U 5	38	Learning medical knowledge means clearly understanding medical concepts.
U 6	39	I think understanding is important in learning medicine.
SNW 1	40	Learning medicine means expanding my medical knowledge and visions.
SNW 2	41	Learning medicine means letting me to see medicine using a new standpoint.
SNW 3	42	Learning medicine means changing my way of seeing the medical phenomena.
SNW 4	43	Learning medicine means finding a better way to see medicine.
SNW 5	44	I can learn more ways of thinking about medicine by learning medicine.
SNW 6	45	Learning medicine means finding a more reasonable way to account for a medical condition.
SA 1	46	Learning medicine means learning how to study systematically, such as using a concept map.
SA 2	47	Learning medicine means learning how to summarize the key points.
SA 3	48	One of the important points in learning medicine is to deliberate about the causes and outcomes of an event.
SA 4	49	The major purpose of learning medicine is to think about the procedure and make a judgment.
SA 5	50	Learning medicine means learning logical deduction in medicine.
SA 6	51	Learning medicine helps me acquire abilities to use inductive analysis.
C 1	52	Learning medicine means learning how to cooperate with others as a team to complete the task.
C 2	53	The major purpose of learning medicine is to learn how to communicate with patients and interact with people.
C 3	54	The major purpose of learning medicine is to learn how to explain and communicate medical conditions to patients and/or family members.
C 4	55	The major purpose of learning medicine is to learn how to cooperate with other medical professionals to solve problems.
C 5	56	The major purpose of learning medicine is to facilitate a good doctor-patient relationship.

Abbreviation List: *M* Memorizing, *PT* Preparing for Testing, *PTP* Practicing Tutorial Problems, *IOK* Increasing One’s Knowledge, *A* Applying, *U* Understanding, *SNW* Seeing in a New Way, *SA* Skills Acquisition, *C* Communication

## Appendix 2

**Table 5:** The Modified Approaches to Learning Medicine (mALM) questionnaire

			No.	Items
Deep Strategy	DS 1	RI 1	1	When learning medicine, I try to relate what I have learned in one subject to what I have learned in others.
	DS 2	RI 2	2	When learning medicine, I like to create a new plausible theory for helping me to summarize a lot of disorganized content.
	DS 3	RI 3	3	When learning medicine, I try to find out the relationships between what I have learned.
	DS 4	RI 4	4	When learning new subjects in medicine, I try to relate to those I have learned.
	DS 5	U 1	5	When reading medical textbooks, I try to understand the meaning of the content.
	DS 6	U 2	6	When learning medicine, I try to understand the content of the medical courses.
	DS 7	U 3	7	When learning medicine, I use systemic ways to learn.
	DS 8	U 4	8	When learning medicine, I read original textbooks or use online resources for better understanding of the content.
	SS 1	MSS 1	9	When learning medicine, the items not tested in the exams are meaningless to me.
	SS 2	MSS 2	10	When learning medicine, I spend as little time on studying medicine as I can, as long as I feel that I can pass the exams. There are many other interesting things to do.
	SS 3	MSS 3	11	When learning medicine, I find out the contents which are worth spending time to study simply because I do not want to spend time and energy on the unrelated content.
Surface Strategy	SS 4	MSS 4	12	When learning medicine, we do not need to be familiar with all the contents simply because there are so many exams we need to pass, and so many subjects we need to learn.
	SS 5	MSS 5	13	When learning medicine, I hope that the teacher can give us what will be tested in the exams, for us to better prepare for the exams.
	SS 6	M 1	14	When learning medicine, I practice rote memorization of the content until I firmly memorize all of the content.
	SS 7	MSS 6	15	I feel that the best way to get good grades in medical exams is the rote the answers of similar questions.
	SS 8	MSS 7	16	I find that to memorize the content of medical subjects by rote makes me achieve good grades on exams, rather than to understand them.
	SS 9	M 2	17	When learning medicine, I am very focused on those which will be tested in exams, and I memorize them by rote.
	SS 10	M 3	18	When learning medicine, I use lecture notes and aids for National Licensing Exams as important learning guides.
	SS 11	M 4	19	When learning medicine, I try to improve my memorization by repeated rote.

Abbreviation List: *DS* Deep Strategy, *SS* Surface Strategy, *RI* Relating Ideas, *U* Understanding, *MSS* Minimizing Scope of Study, *M* Memorization

## Abbreviations

AVE: average variance explained
CFA: confirmatory factor analysis
CFI: comparative fit index
COLM: Conceptions of Learning Medicine
CR: composite reliability
GFI: goodness of fit index
mALM: modified Approaches to Learning Medicine
NFI: normed fit index
RMSEA: root-mean-square error of approximation
SEM: structural equation modeling
